# Optimization of Basophil Activation Test in the Diagnosis and Qualification for Allergen-Specific Immunotherapy in Children with Respiratory Allergy to the House Dust Mite *Dermatophagoides pteronyssinus*

**DOI:** 10.3390/ijms25189959

**Published:** 2024-09-15

**Authors:** Radoslaw Spiewak, Aleksandra Gregorius, Grzegorz Ostrowski, Ewa Czarnobilska

**Affiliations:** 1 Department of Experimental Dermatology and Cosmetology, Jagiellonian University Medical College, ul. Medyczna 9, 30-688 Krakow, Poland; 2 Dermatology & Allergy Practice ‘Dermatolog.eu’ Professor Radoslaw Spiewak, 30-433 Krakow, Poland; 3 Centre of Clinical and Environmental Allergology, Jagiellonian University Medical College, 31-503 Krakow, Poland; ewa.czarnobilska@uj.edu.pl

**Keywords:** allergy diagnosis, in vitro tests, basophil activation test, house dust mites, *Dermatophagoides pteronyssinus*

## Abstract

The aim of this study was to optimize a basophil activation test in the detection of allergy to the house dust mite *Dermatophagoides pteronyssinus* in children with allergic respiratory diseases. This study involved 32 cases, 13 girls and 19 boys aged 4–17 years, with perennial asthma or allergic rhinitis caused by *D. pteronyssinus*. The control group consisted of 13 girls and 19 boys aged 4–17 years with seasonal allergic asthma or rhinitis provoked by Timothy or birch pollen. House dust mite (HDM) allergy was excluded in the controls based on their medical history, skin prick test (SPT) results and sIgE determination. In all patients, a basophil activation test (BAT) was performed with five dilutions of *D. pteronyssinus* allergen (the dilution series ranged from 22.5 to 0.00225 ng/mL). The results were analyzed by using the receiver operating characteristics (ROC) to determine the optimal allergen concentrations, outcome measures and cut-off points that would differentiate most accurately between HDM-allergic and non-allergic patients. As a “gold standard”, criteria for allergen-specific immunotherapy with *D. pteronyssinus* or respective pollens were applied by an experienced pediatric allergist following the guidelines of the European Academy of Allergy and Clinical Immunology. The highest diagnostic efficiency was yielded by the protocol assuming a cut-off value of 9.76% activated basophils after activation with a single allergen concentration of 2.25 ng/mL (sensitivity 90.6%, specificity 100%). This protocol yielded 3 (4.7%) misclassifications, all false negative, when compared with the “gold standard”. There was a strong correlation with the BAT results at 22.5, 2.25 and 0.225 ng/mL (respectively r = 0.90 and r = 0.78, *p* < 0.001), as well as between the BAT at 2.25 ng/mL and SPT (r = 0.82, *p* < 0.001) and between the SPT and sIgE levels (r = 0.78, *p* < 0.001). High cross-reactivity between *D. pteronyssinus* and *D. farinae* was confirmed based on the BAT at 22.5 ng/mL (r = 0.82, *p* < 0.001). In conclusion, the BAT showed very good concordance with the result of a meticulous process of decision-making that combined validated allergy tests (SPT, sIgE) with expert guidelines, specialist knowledge and experience. Facing the risk of the incorrect qualification of patients for costly, long-lasting and potentially risky allergen-specific immunotherapy, the inclusion of a basophil activation test into diagnostic process seems fully justified.

## 1. Introduction

Allergic diseases affect as many as one billion people globally. More than 30% of them suffer from allergic rhinitis (AR) and 20% from asthma, making these diseases a major public health concern [1]. Children are especially vulnerable, with respiratory allergies affecting their physical and mental development. A recent meta-analysis has put the overall prevalence of physician-diagnosed AR in children at 10.48%, while the one-year prevalence of self-reported symptoms consistent with AR amounted to 18.12% and the lifetime prevalence to 19.93%. The prevalence of physician-diagnosed AR in children increased from 8.39% in the period 2012–2015 to 19.87% in 2016–2022 [2]. The prevalence of asthma among children in the USA fluctuated in the past two decades between 7.0 and 9.6% [3]. Of all respiratory allergens, allergy to house dust mites (HDM) seem most prevalent among children; it also is a relevant risk factor for allergic rhinitis and asthma. In an unselected population of four-year-old children from the Isle of Wight, 11.9% were allergic to HDM, which was the most frequent allergen; moreover, being allergic to HDM was a significant risk factor (OR = 8.07) for asthma [4]. In a Portuguese population-based cohort, children with early and persistent HDM allergy were at highest risk for developing asthma (OR = 14.4) and rhinitis (OR = 7.3) [5]. Also in a French cohort, children strongly sensitized to house dust mite were at highest risk of asthma and allergic rhinitis [6]. Sensitization to HDM determines the persistence of childhood asthma, along with the presence of allergic rhinitis [7]. Altogether, these data show the impact of house dust mite allergy and the importance of its early and accurate diagnosis in children.

Allergen-specific immunotherapy (ASIT; allergy vaccination) is the mainstay of causative treatment in allergic asthma and rhinitis. Although more than 35 groups of Dermatophagoides allergens have been identified to date, three allergens—Der p 1, Der p 2 and Der p 23—are considered the major allergens that sensitize the majority of HDM-allergic patients and are relevant to their disease [8]. Approximately 75% of HDM-allergic patients react to Der p 23, with a considerable subgroup reacting solely to this allergen. Similar to Der p 1 and Der p 2, Der p 23 also induces IgE-dependent basophil activation, which confirms its high allergenic activity [9]. Underrepresentation of the major allergens in HDM allergy vaccines may result in low therapeutic efficacy [10]. This underscores the importance of the standardization of HDM allergy vaccines so that they contain all allergens of relevance to a given population. At present, there are considerable differences between manufacturers, as well as between the USA and EU legislation with regard to standardization, including the methodology of potency determination and potency units [11]. This also hints to the importance of component-resolved diagnosis, which helps in selecting allergy vaccines that contain all allergens of relevance to a given patient [8]. 

At present, HDM allergy vaccines are available as allergen extracts or allergoids (i.e., chemically modified allergens) for subcutaneous (SCIT) or sublingual (SLIT) immunotherapy [12]. The ongoing research is focused on the possible applications of recombinant allergens and new biomarkers, as well as novel administration routes of allergy vaccines (oral, intralymphatic, epicutaneous and intradermal) [12,13,14,15]. The interaction between HDM and the skin is another area of research with three major directions: (a) exploring and preventing HDM sensitization via the skin, which would subsequently lead to respiratory manifestations of allergic rhinitis and asthma as a part of the atopic march [16]; (b) skin sensitization to HDM manifesting clinically as extrinsic atopic dermatitis (i.e., eczema triggered by exogenous factors with the involvement of specific immunological hypersensitivity) [17]; (c) the applicability of HDM ASIT in the treatment of patients with extrinsic atopic dermatitis [18]. While the therapeutic efficacy and safety of SCIT and SLIT with HDM are well-documented and widely accepted in asthma and allergic rhinitis [19,20,21], there is also growing evidence that SIT may be beneficial in patients with atopic dermatitis due to relevant mite allergies [22,23].

The effectiveness of ASIT largely depends on the proper qualification and choice of the right allergen composition of the vaccines that would correspond with the patients’ sensitization profiles [24,25]. Assessing the relevance of HDM allergy may be challenging to doctors while differentiating between mite-induced and mite-sensitized rhinitis and asthma. In the first instance, patients have episodes of disease following exposure to HDM allergens; in the second, patients happen to have positive allergy tests to HDM yet there is no clear relation between HDM exposure and symptoms [26,27]. History-taking and clinical observations may prove insufficient; in some cases, nasal or bronchial provocations are necessary [28]. Despite confirmed correlations with the clinical symptoms and intensity of inflammation, the specific IgE (sIgE) levels are not sufficient to distinguish between silent sensitization and clinically relevant allergy [29,30]. This again underscores the importance of accurate allergy diagnoses.

Among the recent developments, the basophil activation test (BAT) is among the most promising methods, with much international following and effort invested into its standardization and quality assurance, as well as its official clearance for routine diagnostic use [31,32,33]. In our previous work, we have shown that the BAT can be useful in both qualifying and monitoring ASIT [34,35,36,37]. In this study, we undertook another approach for the assessment of the diagnostic accuracy of the BAT in HDM allergy in children, in which various BAT outcome measures were scrutinized against the arguably most relevant “gold standard”, i.e., the qualification for ASIT carried out by a pediatric allergist following the guidelines of the European Academy of Allergy and Clinical Immunology (EAACI) [38,39,40]. This, in our opinion, has set the bar higher than simply correlating the BAT results with routine diagnostic methods such as the skin prick test (SPT) or sIgE.

## 2. Results

**Figure 1:**
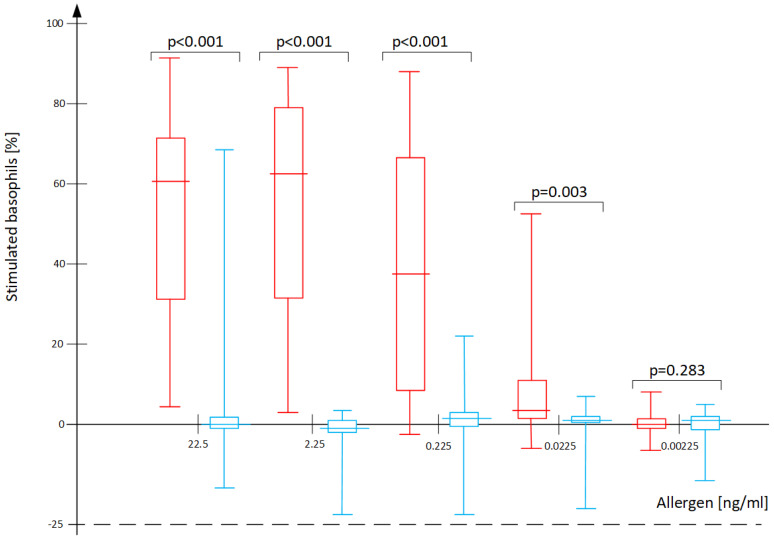
Results of the BAT expressed as the percent of stimulated (CD63+) basophils with different concentrations of *D. pteronyssinus* allergen in cases (red) and controls (blue). Numerical data for the graphs are listed in the Supplementary Materials (Table S1).

**Figure 2:**
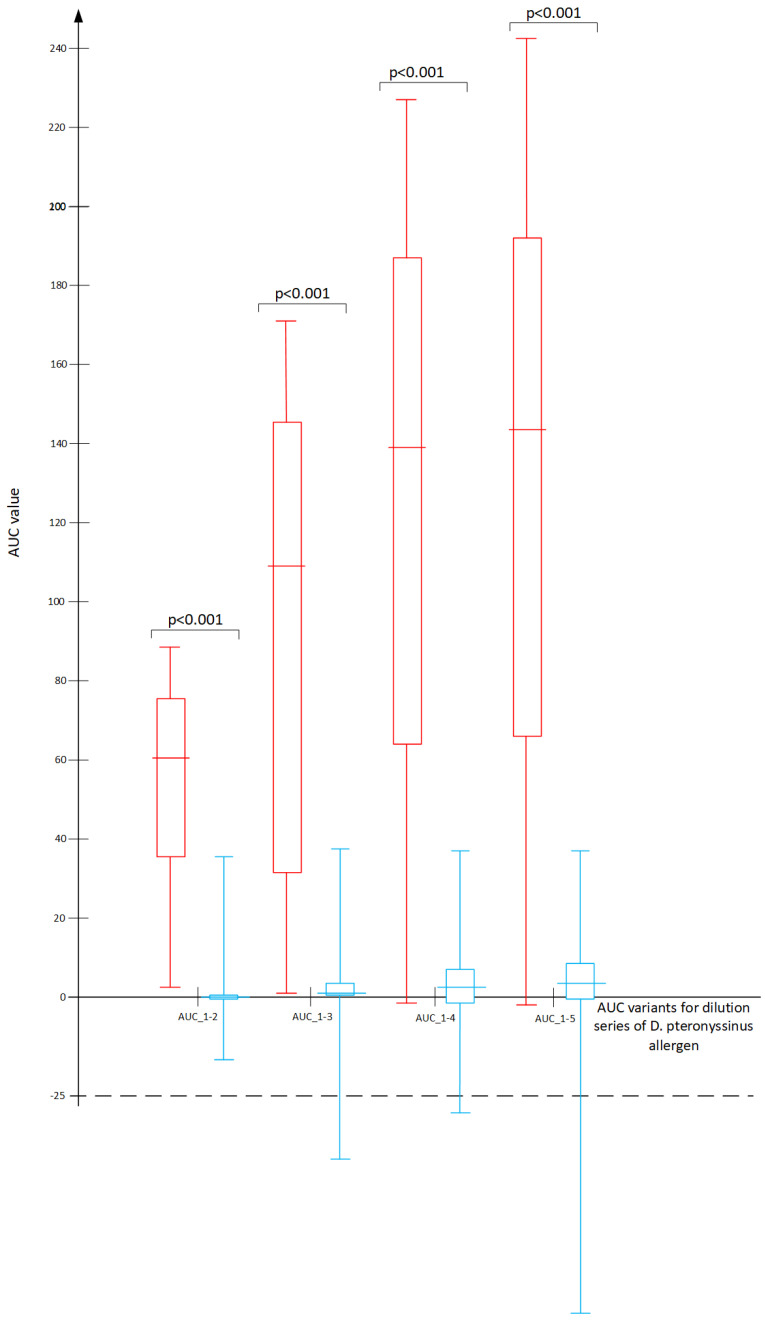
Cumulative results of the BAT (area under the curve—AUC) in cases (red) and controls (blue). Numerical data for the graphs are listed in the Supplementary Materials (Table S2).

In all recruited patients, the basophil activation test (BAT) demonstrated basophil activation in at least one of the two positive control vials but not in the negative control vial, meaning that no patient had to be excluded as a non-responder from our case group or control group. In the initial statistical analysis, no significant differences were observed between younger and older childrens’ subgroups as divided by the median 9.5 years of age. Therefore, the analyses presented in the paper were done for the entire age groups. The median wheal size in response to *D. pteronyssinus* in the SPT was 5.5 mm in the case group and 0 mm in the control group (Mann–Whitney “U” test: *p* < 0.001). The median sIgE to *D. pteronyssinus* in the case group was 30.15 kU/L, while it was 0.08 kU/L in the controls (Mann–Whitney “U” test: *p* < 0.001). There were statistically relevant differences between the case and control groups in the BAT results for the 4 highest concentrations of *D. pteronyssinus* allergens, with the differences disappearing at the lowest concentration (Figure 1). The differences between the cases and controls were highly significant in all tested variants of the AUC models; however, there were considerable overlaps of the individual result ranges, which are represented by the long “whiskers” in Figure 2. A strong cross-reactivity between *D. pteronyssinus* and *D. farinae* was confirmed based on the BAT at the concentration of 22.5 ng/mL for both allergens (r = 0.82, *p* < 0.001).

The receiver operating characteristics (ROC) analysis showed that the highest diagnostic accuracy for all analyzed parameters was achieved with a single BAT with *D. pteronyssinus* allergen at a concentration of 2.25 ng/mL and cut-off value of 9.76%. With such parameters, 95.3% of the patients were accurately qualified by the BAT as either *D. pteronyssinus*-allergic or non-allergic, with a sensitivity rate of 90.63% and specificity rate of 100% when compared with the “gold standard”. The second rank among the test methods was occupied ex aequo by a single-point BAT at 22.5 ng/mL and two AUC models combining results for 2 or 3 allergen concentrations. The complete results of the ROC analyses are shown in Table 1. 

**Figure 3:**
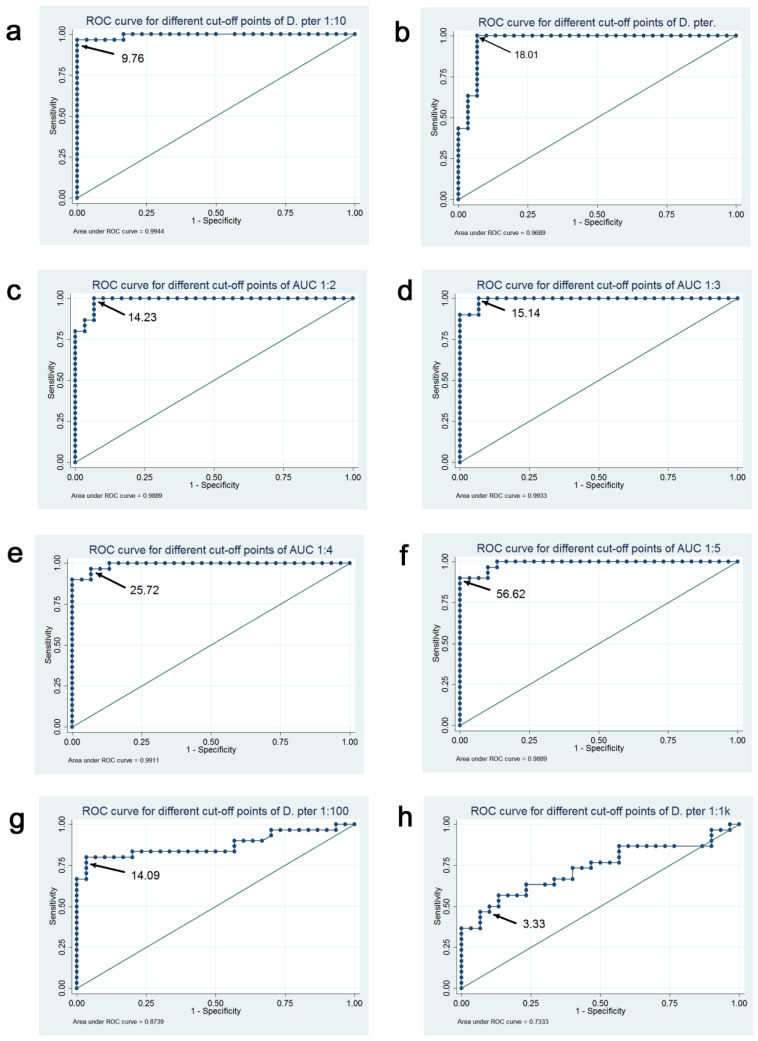
Graphs representing the receiver operating characteristics (ROC) analyses of the diagnostic accuracy of the compared BAT outcomes. The arrows indicate the computed cut-off values. Letters (**a**–**h**) assigned to individual graphs refer to detailed data presented in Table 1.

A visual presentation of the ROC analyses is shown in Figure 3. An ROC curve closely following the diagonal line in the graph would mean that the test is useless at differentiating between those with HDM allergy and those without allergy, while an ROC curve tightly adhering to the left and top frames of the plot would indicate a test that perfectly discriminates between HDM-allergic and non-allergic patients.

In a further analysis of diagnostic accuracy, the BAT results at a *D. pteronyssinus* concentration of 2.25 ng/mL and cut-off value of 9.76% misclassified 3 patients in the case group (sensitized according to the “gold standard”) as “non-sensitized” (Table 2). If the classification has been done with the cut-off recommended by the manufacturer (15%), one more case would have been missed (Table 3). This indicates that the factory settings may require adjustments in order to achieve the most reliable results.

The strongest correlation was observed between the BAT results at 2.25 and 22.5 ng/mL, followed by the correlations between the BAT results at 2.25 ng/mL and the commercial skin prick test results, between the BAT results at 2.25 and 0.225 ng/mL and between the skin prick test and specific IgE level (Table 4). Altogether, these results demonstrated good concordance between the BAT and the routine allergy test results that contributed to the “gold standard” (clinical assessment based on the patients’ history combined with SPT and sIgE results).

## 3. Discussion

The overall impression from the data obtained in this study was of good concordance between the results of the BAT and the “gold standard”—a clinical qualification for ASIT by an expert allergist based on a patient’s history and routine allergy tests. The control group was selected in the study with the goal of providing the maximal similarity between the groups (sex, age, primary disease), except for the fact of being allergic or not to *D. pteronyssinus*. This is similar to day-to-day clinical practice, when a pediatric allergist faces children with similar symptoms and has to decide whether to initiate the specific immunotherapy and lastly to select the relevant allergens for the vaccine. ASIT is a procedure that lasts 3 years or more, requires repeated visits (injections, prescriptions, follow ups), may be costly and is not entirely risk-free. Therefore, a cautious and proper qualification for the procedure is imperative in allergy practice, and every diagnostic tool is welcome that reliably supports the decision-making process.

Despite a growing interest in the BAT among allergists, there are not many studies on the usefulness of the BAT in the qualification or monitoring of ASIT with HDM in children. In a pioneering study in this field, the authors compared 24 mite allergic with 23 atopic children using the BAT (CD63+) with mite extracts. Using the ROC methodology, they found that for stimulation with 16 µg/mL of mite extract and a cut-off value of 18%, the specificity and sensitivity were each 96%, while at 1.6 µg/mL and 8%, the specificity increased to 100% at the expense of the sensitivity decreasing to 82% [41]. In a subsequent study of 53 children with respiratory allergy with SPT used as the “gold standard”, the sensitivity of the BAT (CD63+) was 96.9% and the specificity was 88.9% for *D. pteronyssinus*, with respective values of 89.3% and 100% for *D. farinae* [42]. In another study of 47 pediatric patients with allergic rhinitis to HDM and 15 non-allergic healthy subjects, the BAT test with HDM and a cut-off point of 12.5% CD63+ basophils yielded a sensitivity rate of 90%, specificity rate of 73%, positive predictive value of 0.70 and negative predictive value of 0.91 [42].

In a recent study of children with asthma and rhinitis to *Dermatophagoides farinae*, a house dust mite closely related to *D. pteronyssinus*, the authors compared 39 children aged 6–10 years with allergic rhinitis and asthma who were qualified for ASIT with *D. farinae* with 15 children aged 5–8 years with asthma and rhinitis who were not allergic to *D. farinae* [43]. The authors preformed ROC analyses similar to those presented in our article and found out that the best sensitivity (80.8%) and specificity (100%) were achieved with a *D. farinae* allergen concentration of 10 µg/mL. The authors also demonstrated an inverse correlation (r= –0.706, *p* < 0.001) between the percent of activated basophils in the BAT (CD63+) and the asthma control test outcome (C-ACT; a higher value means better asthma control), as well as a positive correlation (r = 0.693, *p* < 0.001) between the percent of activated basophils and visual analogue scale of rhinitis (a higher value reflects a greater intensity of symptoms). Similar to our results with *D. pteronyssinus*, the authors also demonstrated significant correlations between the BAT results and the SPT with *D. farinae* allergen (r = 0.812, *p* < 0.001) and sIgE level (r = 0.602, *p* < 0.001). Finally, the authors followed the course of immunotherapy with *D. farinae* allergen in 21 children and demonstrated significant decreases in the percentages of activated basophils after 3 years of ASIT [43]. Altogether, the results of the excellent work on *D. farinae* allergy in children are in good concordance with results of our previous study of 21 children receiving ASIT with *D. pteronyssinus* allergen, in whom we repeated the BAT three times during their course of immunotherapy, demonstrating a significant decrease in numbers of stimulated basophils in response to *D. pteronyssinus* at 0.225 ng/mL, along with a decrease in CD-sens values [37]. The cross-reactivity between *D. pteronyssinus* and *D. farinae* is well known [44]. In the present study, we have also confirmed the cross-reactivity between these two *Dermatophagoides* species; in our patients, the correlation between the BAT responses to *D. pteronyssinus* and *D. farinae* allergens at 22.5 ng/mL was very strong (r = 0.82) and relevant (*p* < 0.001).

As with any diagnostic method, the basophil activation test has its limitations. In a study of local allergic rhinitis (LAR) in children under 15 years, 7 patients with LAR reacted to a nasal provocation test but only one of them was positive on the BAT test [45]. LAR is characterized by a lack of circulating sIgE antibodies; therefore, circulating basophils are probably not “armed” with allergen-specific sIgE. In this regard, the BAT is subject to the same limitations as the SPT and sIgE; thus, apparently it would not replace the nasal provocation test with a suspected allergen [46,47]. When starting this study, our expectation was that a complex AUC analysis that would compile BAT responses to a range of allergen concentrations might offer some advantage over the routine single- and two-point measurements. In spite of this, a single-point measurement of basophil stimulation with *D. pteronyssinus* allergen at 2.25 ng/mL proved best in our study settings, followed ex aequo by a single-point BAT at 22.5 ng/mL, then two-point AUC and three-point AUC models. Putting this together, our two- and three-point AUC models performed well but slightly worse than the best single-point result. Given the small differences in the indices and relatively small population size, the AUC models might still deserve another chance in other populations and applications, e.g., in the flow-assisted diagnosis of drug, vaccine or food allergies—areas in which the selection of optimal allergen concentrations for the BAT frequently poses a challenge [48,49,50]. As reductions in BAT reactivity during SIT have been observed in numerous studies, the application of the BAT for monitoring and guiding the therapy is another option deserving further consideration. Depending on the circumstances, a lack of reduction in BAT reactivity could prompt one to either increase the vaccine dosage or abandon the treatment [31]. However, a recent study demonstrated that the BAT may be more useful in monitoring rather than predicting responses to ASIT [51]. Further studies on this topic are needed to resolve this issue. Only a prospective study assessing the final effectiveness of ASIT in relation to the initial BAT results could validate the BAT as a possible method for qualifying patients for ASIT.

## 4. Material and Methods

### 4.1. Study Participants

The case group was recruited among patients routinely qualified for ASIT with house dust mites by an experienced pediatric allergist (EC) in line with the EAACI recommendations [38,39,40]. Among the pediatric patients qualified for routine ASIT, 32 HDM-allergic patients agreed to participate (13 girls and 19 boys aged 4–17 (median 9.5) years). The main complaints of the patients were perennial asthma (15 cases, 47%) or allergic rhinitis (29 cases, 91%) caused by house dust mites (HDM). The causality was confirmed by their medical history combined with positive SPT and sIgE results for *D. pteronyssinus*. The control group consisted of identical numbers of 13 girls and 19 boys aged 4–17 (median 9.5) years with seasonal allergic asthma (11 controls, 34%) or rhinitis (30 controls, 94%) provoked by timothy pollen (18 controls, 56%), birch pollen (12 controls, 38%) or both (2 controls, 6%). We aimed to recruit controls with clinical and demographic profiles as close to the cases as possible, except the HDM allergy, which was excluded in the controls based on their medical history and SPT and sIgE results. The BAT was carried out only after the completion of the qualification process for ASIT; therefore, the results did not influence the pediatric allergist’s decisions. Researchers who performed the BAT on the patients’ blood samples (RS, AG) handled all samples according to one protocol—not knowing the allergy status of the patients. Ethical clearance was obtained for this study from the University’s Bioethics Committee. Informed consent was obtained from all participants and their legal guardians.

### 4.2. Allergy Tests

#### 4.2.1. Routine Allergy Tests

Skin prick tests (SPT) were performed on the ventral forearm with an allergen of *Dermatophagoides pteronyssinus* (Allergopharma, Reinbek, Germany), positive control (histamine 1 mg/mL) and negative control (physiological saline). The result was considered positive if the diameter of the wheal reaction to both *D. pteronyssinus* and histamine was at least 3 mm bigger than the diameter of the negative control. Specific IgE levels against *D. pteronyssinus* in the cases and controls, as well as Timothy and birch pollens in controls, were measured using UniCAP 100 (Phadia, Uppsala, Sweden), with sIgE levels of 3.5 kUI/L or higher considered positive.

#### 4.2.2. Basophil Activation Test

**Figure 4:**
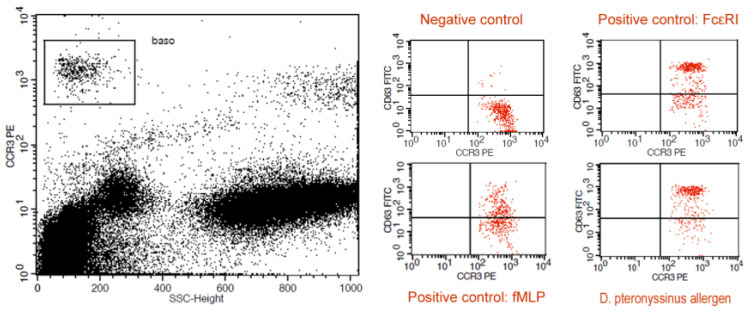
Gating strategy for basophils (**left**) and exemplary graphs showing basophil reactivity to controls and response to the allergen of *D. pteronyssinus* (**right**).

The basophil activation test (BAT) is a functional ex vivo, in vitro assay that measures the degree of basophil activation in response to allergens or controls. The activation of basophils was measured as the percent of basophils expressing the CD63 marker in the blood samples tested using flow cytometry [33]. The test was carried out in anticoagulant (EDTA)-treated blood samples. The tests were started no later than 2 h after taking blood. Commercial, IVD-certified test kits from Bühlmann Laboratories (Schönenbuch, Switzerland) were used. Two positive controls were used—monoclonal antibodies against the IgE membrane receptor (FcεRI), which initiate basophil activation by the FcεRI, and the bacterial polypeptide N-formylmethionyl-leucyl-phenylalanine (fMLP), which causes the unspecific degranulation of basophils. A dilution buffer served as the negative control. The manufacturer’s protocol was modified using a dilution series of five 10-fold dilutions of *D. pteronyssinus* allergens, rather than a single 5-fold allergen dilution as recommended by the manufacturer. The idea behind this modification was to obtain a broader scope of the allergen’s concentration to calculate the area under the curve indices (AUC; see below). The dilution series included concentrations of 22.5 ng/mL, 2.25 ng/mL, 0.225 ng/mL, 0.0225 ng/mL and 0.00225 ng/mL. In order to check for possible cross-reactivity, basophil degranulation was tested in response to allergens of another ubiquitous house dust mite, *D. farinae,* at a single concentration of 22.5 ng/mL. Blood samples were stained with fluorescein-coupled monoclonal antibodies against the surface antigen CD63 (anti-CD63-FITC) and a phycoerythrin-coupled antibody against the human chemokine receptor CCR3 (anti-CCR3-PE). The samples were incubated with the antibodies for 15 min at 37 °C, with subsequent lysis to remove the erythrocytes. The flow cytometry was carried out using a FacsCalibur flow cytometer (Becton, Dickinson and Co., Franklin Lakes, NJ, USA) with the accompanying software BD CellQuest™ Pro ver. 6.0 (BD Biosciences, San Jose, CA, USA). The basophils were gated as CCR-positive cells with low side scatter (CCR3+, SSC^low^). The CD63-positive cells (CD63+) were counted among 500 cells in this subpopulation. Exemplary graphs of the gated basophils’ reactions to negative and positive controls, as well as the *D. pteronyssinus* allergen, are shown in Figure 4.

### 4.3. Statistical Analysis

The statistical analyses were carried out using the packages Stata 11.2 (StataCorp LP, College Station, TX, USA) and Statistica 10 (StatSoft, Tulsa, OK, USA), with the significance level set at *p* ≤ 0.05. The main focus was possible differences in the BAT results with HDM allergens between cases with confirmed allergy to *D. pteronyssinus* and controls allergic to other allergens. For each measured variable, the minimum, maximum, median and interquartile range were computed. Due to the marked skewness of all variables, a non-parametric Mann–Whitney “U” test was applied to check for relevant differences. The results at the different allergen concentrations in the dilution series were analyzed both individually and collectively as the area under the curve (AUC). The AUC compiles a series of results—in this case the percentages of activated basophils in response to different concentrations of allergen—into one numerical value (test result) that is easier to manipulate mathematically and correlate with clinical symptoms. Additionally, establishing cut-off values for one variable seems most practical in clinical settings. The idea of implementing the AUC was based on an assumption that basophils of individual patients may react with a maximal stimulation to various allergen concentrations; thus, using a range of allergen concentrations would result in a better chance for the detection of a maximal basophil response to the allergen in a patient. Furthermore, combining all results in the range into one numerical AUC value would enable easy interpretation, e.g., applying one cut-off value for the entire range. Whether the AUC would indeed offer any advantage and what would be the optimal range of allergen concentrations were among the research questions in this study. 

In order to find the optimal cut-off values that would best differentiate between sensitized and non-sensitized patients, the receiver operating characteristics (ROC) analyses were carried out. The accuracy was assessed using the values for the sensitivity and specificity [52,53]. A sensitivity rate of 100% would mean that all sensitized patients would be correctly classified as sensitized. A specificity rate of 100% would mean that all non-allergic persons would be classified by the test as non-allergic [54,55]. In the ROC analysis, the predictors under investigation were (1) the results of the BAT at each individual allergen concentration (22.5 ng/mL, 2.25 ng/mL, 0.225 ng/mL, 0.0225 ng/mL) and (2) the above-mentioned AUC values. The outcome here was the percentage of correctly (according to “gold standard”) classified patients. The AUC values were calculated for 2, 3, 4 or all 5 allergen concentrations in the dilution series, always including the highest concentration and adding stepwise the lower ones. They were designated respectively as BAT AUC_1-2, BAT AUC_1-3, BAT AUC_1-4 and BAT AUC_1-5. Additionally, a correlation analysis was carried out between the BAT results and SPT wheal responses and sIgE levels against *D. pteronyssinus*. Correlations between various outcome measures were also analyzed.

The “gold standard” used as the reference in this study was the qualification of a child for ASIT carried out by an experienced pediatric allergist (EC) who followed the guidelines of the EAACI [38,39,40]. The case group consisted of children suffering from allergic rhinitis or asthma qualified for ASIT with *D. pteronyssinus* allergens (relevant respiratory allergy to HDM). The control group consisted of children suffering from allergic rhinitis or asthma qualified for ASIT with birch or timothy pollen allergens but with no HDM allergy (relevant respiratory allergy to non-HDM allergens, negative SPT and sIgE, no complaints related to house dust).

## 5. Conclusions

In the present study, we opted for an assessment of the diagnostic accuracy of the BAT in children with HDM allergy, in which a range of BAT outcomes were scrutinized against the relevant “gold standard” of qualification for ASIT carried out by a pediatric allergist following the guidelines endorsed by the EAACI. With such an approach, instead of merely pronouncing a patient “allergic to HDM” or “not allergic to HDM” (as in the case of the SPT or sIgE), the allergist must carry out the complex process of deciding the clinical relevance of the sensitization, i.e., its causative role in the patient’s illness (worsening upon specific allergen exposure and improvement after avoidance) and the probability of health benefits from ASIT with HDM allergens. Very good concordance between the BAT as a relatively well standardized in vitro method and complex expert assessments makes the BAT something more than “just another in vitro method”. With this statement, we are not suggesting that the BAT would replace a complex qualification for ASIT performed by an experienced allergist. Nevertheless, it showed very good concordance with the result of a meticulous process of decision-making that combined validated allergy tests (SPT, sIgE) with expert guidelines, specialist knowledge and experience. In our opinion, the “gold standard” used in this work was demanding, compared groups were very similar clinically and the test itself performed rather well with the fine-tuned parameters. Facing the risk of the incorrect qualification of patients for costly, long-lasting and potentially risky allergen-specific immunotherapy, the inclusion of the basophil activation test into diagnostic process seems fully justified.

## Figures and Tables

**Table 1:** A receiver operating characteristics (ROC) analysis of the diagnostic accuracy of the compared parameters.

Analyzed BAT Results	Optimal Cut-Off	Correctly Classified (%)	Sensitivity (%)	Specificity (%)	AUC for ROC (95% CI)	Plot in Figure 3
BAT *D. pteronyssinus* 2.25 ng/mL	9.76%	95.31	90.63	100.00	0.98 (0.95–1.00)	a
BAT *D. pteronyssinus* 22.5 ng/mL	18.01%	93.75	93.75	93.75	0.96 (0.92–1.00)	b
BAT AUC_1-2	14.23	93.75	93.75	93.75	0.98 (0.96–1.00)	c
BAT AUC_1-3	15.14	93.75	93.75	93.75	0.97 (0.94–1.00)	d
BAT AUC_1-4	25.72	92.19	90.63	93.75	0.96 (0.91–1.00)	e
BAT AUC_1-5	12.79	92.19	96.88	87.50	0.96 (0.91–1.00)	f
BAT *D. pteronyssinus* 0.225 ng/mL	14.09%	85.94	75.00	96.88	0.84 (0.73–0.95)	g
BAT *D. pteronyssinus* 0.0225 ng/mL	3.33%	70.31	56.25	84.38	0.72 (0.59–0.85)	h

**Table 2:** Diagnostic accuracy of the BAT with *D. pteronyssinus* allergen at 2.25 ng/mL when using a cut-off value of 9.76% as computed in the present ROC analysis.

“Gold Standard”	BAT-Positive	BAT-Negative
Relevant allergy to *D. pteronyssinus*	29	3
No allergy to *D. pteronyssinus*	0	32

**Table 3:** Diagnostic accuracy of the BAT with *D. pteronyssinus* allergen at 2.25 ng/mL when using a cut-off value of 15% as recommended by the manufacturer.

“Gold Standard”	BAT-Positive	BAT-Negative
Relevant allergy to *D. pteronyssinus*	28	4
No allergy to *D. pteronyssinus*	0	32

**Table 4:** Correlations between measured responses to *D. pteronyssinus* allergens based on SPT, sIgE and BAT results at various concentrations. The strongest correlations are marked in bold typeface.

	sIgE *D. pteronyssinus* [kU/L]	SPT *D. pteronyssinus* (Wheal Diameter) [mm]	BAT *D. pteronyssinus* 22.5 ng/mL (% act.)	BAT *D. pteronyssinus* 2.25 ng/mL (% act.)	BAT *D. pteronyssinus* 0.225 ng/mL (% act.)	BAT *D. pteronyssinus* 0.0225 ng/mL (% act.)
SPT *D. pteronyssinus* (wheal diameter) [mm]	**r = 0.78 ** ***p* < 0.001**					
BAT *D. pteronyssinus* 22.5 ng/mL (% act.)	r = 0.71 *p* < 0.001	r = 0.77 *p* < 0.001				
BAT *D. pteronyssinus* 2.25 ng/mL (% act.)	r = 0.74 *p* < 0.001	**r = 0.82 ** ***p* < 0.001**	**r = 0.90 ** ***p* < 0.001**			
BAT *D. pteronyssinus* 0.225 ng/mL (% act.)	r = 0.55 *p* < 0.001	r = 0.64 *p* < 0.001	r = 0.67 *p* < 0.001	**r = 0.78 ** ***p* < 0.001**		
BAT *D. pteronyssinus* 0.0225 ng/mL (% act.)	r = 0.39 *p* = 0.001	r = 0.39 *p* = 0.001	r = 0.44 *p* < 0.001	r = 0.56 *p* < 0.001	r = 0.74 *p* < 0.001	
BAT *D. pteronyssinus* 0.00225 ng/mL (% act.)	r = 0.06 *p* = 0.666	r = −0.16 *p* = 0.210	r = −0.03 *p* = 0.830	r = 0.05 *p* = 0.677	r = 0.15 *p* = 0.238	r = 0.34 *p* < 0.001

## Data Availability

The original contributions presented in the study are included in the article/supplementary material, further inquiries can be directed to the corresponding author.

## Supplementary Materials

The following supporting information can be downloaded at https://www.mdpi.com/article/10.3390/ijms25189959/s1.
