# Supplementary material for: Optimization of Basophil Activation Test in the Diagnosis and Qualification for Allergen-Specific Immunotherapy in Children with Respiratory Allergy to the House Dust Mite Dermatophagoides pteronyssinus
Source: Int J Mol Sci. 2024 Sep 15;25(18):9959. doi: 10.3390/ijms25189959 (PMC11432158; doi:10.3390/ijms25189959)
Supplement: Supplementary file 1 [file ijms-25-09959-s001.zip › ijms-3211694-supplementary.pdf]

Optimization of basophil activation test in the diagnosis and qualification for allergen-specific immunotherapy in children with respiratory allergy to house dust mite Dermatophagoides pteronyssinus

Supplementary material

Table S1. Results of BAT (percent of stimulated basophils) at different concentrations of D. pteronyssinus allergen in compared groups. A graphical presentation of these data is in Figure 1 of the main article.

Allergen concentration	% of activated basophils*	Cases	Controls
22.5 ng/ml	Minimum	4.86	−16.10
	Q1	32.46	−1.02
	Median	61.43	−0.03
	Q3	72.83	2.03
	Maximum	92.09	68.34
	Mann Whitney “U”	p<0.001	
2.25 ng/ml	Minimum	−0.30	−22.08
	Q1	31.64	−1.23
	Median	62.89	−0.43
	Q3	79.22	0.71
	Maximum	89.10	3.54
	Mann Whitney “U”	p<0.001	
0.225 ng/ml	Minimum	−2.67	−21.87
	Q1	8.61	−0.14
	Median	37.61	1.26
	Q3	66.59	2.86
	Maximum	87.38	20.26
	Mann Whitney “U”	p<0.001	
0.0225 ng/ml	Minimum	−5.48	−21.20
	Q1	0.22	0.32
	Median	4.15	0.82
	Q3	10.94	1.60
	Maximum	52.32	7.12
	Mann Whitney “U”	p=0.003	
0.00225 ng/ml	Minimum	−6.77	-14.12
	Q1	−1.12	−0.60
	Median	−0.10	0.32
	Q3	1.47	1.88
	Maximum	8.00	5.04
	Mann Whitney “U”	p=0.283	

* Q1, lower quartile (25th percentile); Q3, upper quartile (75th percentile)

Table S2. Cumulative results of BAT (area under the curve, AUC) in compared groups. A graphical presentation of these data is in Figure 2 of the main article.

Allergen concentration	% of activated basophils*	Cases	Controls
AUC_1-2	Minimum	2.28	−19.09
	Q1	35.50	−0.87
	Median	60.91	−0.13
	Q3	75.14	0.59
	Maximum	88.41	35.56
	Mann Whitney "U"	p<0.001	
AUC_1-3	Minimum	0.87	−41.07
	Q1	61.81	0.74
	Median	109.28	0.97
	Q3	145.61	3.82
	Maximum	170.80	37.75
	Mann Whitney "U"	p<0.001	
AUC_1-4	Minimum	−1.55	−62.60
	Q1	64.25	−1.35
	Median	139.39	2.54
	Q3	187.18	7.38
	Maximum	227.52	37.94
	Mann Whitney "U"	p<0.001	
AUC_1-4	Minimum	−2.60	-80.26
	Q1	65.90	−0.41
	Median	143.72	3.36
	Q3	192.06	8.11
	Maximum	242.46	37.25
	Mann Whitney "U"	p<0.001	

* Q1, lower quartile (25th percentile); Q3, upper quartile (75th percentile)

AUC_1-2 (area under the curve calculated for 2 highest allergen concentrations)

AUC_1-3 (area under the curve calculated for 3 highest allergen concentrations)

AUC_1-4 (area under the curve calculated for 4 highest allergen concentrations)

AUC_1-5 (area under the curve calculated for 5 highest allergen concentrations)
